# Myofascial trigger points as a primary cause of equine lameness: a biomechanical, neurophysiological, and fascial review

**DOI:** 10.3389/fvets.2026.1852775

**Published:** 2026-05-20

**Authors:** Markus Scheibenpflug, Kevin K. Haussler

**Affiliations:** 1 Equinelibrium – Veterinary Biomechanics Practice, Leuggern, Switzerland; 2 College of Veterinary Medicine, Lincoln Memorial University, Harrogate, TN, United States

**Keywords:** biotensegrity, diagnostic protocol, dry needling, equine lameness, fascial chains, gait asymmetry, Lyme borreliosis, myofascial pain syndrome

## Abstract

Equine lameness diagnosis is dominated by a joint- and tendon-centric paradigm. The standard diagnostic algorithm relies on gait observation, perineural and intrasynovial anesthesia, and cross-sectional imaging. It is directed almost exclusively at skeletal and articular structures. Myofascial trigger points (MTrPs) are hypersensitive, hyperirritable loci within taut bands of skeletal muscle. They produce local and referred pain on compression or contraction. In horses, MTrPs are a clinically relevant but systematically overlooked source of primary lameness. This review synthesises evidence from equine and comparative research. We argue that MTrPs can act as the primary—not merely secondary—cause of gait asymmetry, performance deficits, and pain behavior in horses. Key mechanisms include the energy crisis model of MTrP formation, peripheral and central sensitization, referred pain projection mimicking distal limb pathology, and the biomechanical consequences of MTrP-induced muscle inhibition on gait. Electrophysiological studies confirm that equine MTrPs show the same spontaneous electrical activity as human and animal MTrPs. Prevalence studies indicate that MTrPs are present in many sport horses. Dressage horses show particularly high prevalence in the cervical and thoracolumbar musculature. Of clinical importance is the referred pain phenomenon: MTrPs in proximal muscles such as the gluteus medius, longissimus lumborum, and biceps femoris may produce apparent distal limb pain. Such pain can be clinically indistinguishable from joint or tendon pathology. This has direct and underappreciated implications for diagnostic accuracy. We propose that systematic myofascial palpation should be integrated into the routine equine lameness workup as a first-tier diagnostic step. Rapid resolution of gait asymmetry following targeted MTrP treatment should be interpreted as supportive evidence of myofascial origin. We further delineate the clinically relevant trigger point activity spectrum from active to latent states, address differential diagnoses such as Lyme-associated diffuse myalgia and primary myopathies (PSSM2, MIM, IMM), summarise the principal therapeutic modalities used in equine MTrP management, and examine biotensegrity as a mechanobiological framework explaining the multisegmental fascial effects of myofascial dysfunction.

## Highlights

- MTrPs are present in a substantial proportion of sport horses and are frequently underdiagnosed.
- Peripheral sensitization and referred pain from MTrPs may mimic distal orthopaedic lameness.
- In horses, MTrPs can cause signs of lameness independent of joint, tendon, or ligament pathology.
- Standard diagnostic local anesthesia does not identify myofascial pain as a lameness source.
- Latent MTrPs can impair motor control without producing overt signs of pain or lameness.
- Borreliosis may cause diffuse myalgia distinct from MPS-associated lameness.
- Biotensegrity may link MTrPs to widespread myofascial and kinematic disruption.
- Myofascial examination should be integrated into routine equine lameness assessment.

## 1 Introduction

The diagnosis and treatment of equine lameness represents one of the most significant challenges in equine veterinary practice, with musculoskeletal disorders accounting for most performance limitations and welfare concerns in sport horses (1). The conventional diagnostic framework is hierarchically structured: visual lameness assessment, followed by perineural and intrasynovial anesthesia to localize pain to specific anatomical regions, followed by radiography, ultrasonography, computed tomography, or magnetic resonance imaging to identify structural pathology. This framework has driven substantial advances in the diagnosis and treatment of articular, osseous, and tendinous conditions. However, it contains a systematic blind spot: it does not account for general or localized myofascial pain as a primary source of lameness or poor performance.

Myofascial pain refers to pain arising from skeletal muscle and its associated fascial structures, and may present as either localized or diffuse dysfunction affecting movement and performance. It is increasingly recognized as a relevant component of musculoskeletal disorders across species, yet it remains poorly integrated into equine lameness evaluation, where diagnostic emphasis is primarily placed on articular and tendinous structures. Within this context, focal manifestations become particularly relevant, most notably myofascial trigger points (MTrPs).

MTrPs are defined as hypersensitive, hyperirritable loci within taut bands of skeletal muscle that produce characteristic local twitch responses, referred pain patterns, and autonomic phenomena on palpation or needling (2). In human medicine, MTrPs are recognized as a leading cause of musculoskeletal pain and a primary contributor to chronic myofascial pain syndromes (3). In small animal veterinary medicine, MTrPs have been identified as sources of lameness, reduced range of motion, and performance deficits (4). In horses, however, MTrPs remain largely absent from standard diagnostic and treatment algorithms, despite electrophysiological evidence confirming their existence and clinical characteristics comparable to those documented in other species (5).

This review argues for a paradigm shift in equine lameness diagnosis: from the current orthopaedic-centric model to one that systematically includes myofascial examination as a first-tier diagnostic step. We present the neurophysiological basis of MTrP formation and referred pain, review current equine-specific evidence, analyze the biomechanical mechanisms by which MTrPs produce gait asymmetry, and outline the clinical, diagnostic, and therapeutic implications of myofascial pain as a primary lameness diagnosis.

### 1.1 Literature search strategy

A narrative review of the literature was conducted using PubMed/MEDLINE, Web of Science, and Scopus. Search terms included combinations of: ‘myofascial trigger point’, ‘equine’, ‘horse’, ‘lameness’, ‘referred pain’, ‘myofascial pain’, ‘dry needling’, ‘biotensegrity’, ‘fascial network’, ‘Lyme borreliosis’, ‘central sensitization’, ‘peripheral sensitization’, ‘muscle inhibition’, and ‘gait asymmetry’. Searches were not restricted by date; the most recent search was conducted in March 2026. Inclusion criteria were: original research articles, systematic reviews, or consensus statements pertaining to myofascial pain, trigger points, equine musculoskeletal pain, fascial biomechanics, or directly applicable comparative evidence from human or veterinary medicine. Case reports without quantitative outcome data were excluded. References cited in retrieved articles were examined for additional relevant sources. A total of 58 references are cited in this review; given the novelty of myofascial pain as a primary lameness diagnosis in equine practice, comparative human and animal literature was included wherever equine-specific data were absent.

## 2 Myofascial trigger points: definition, formation, and classification

### 2.1 Definition and clinical characteristics

The MTrP was first systematically characterized by Simons et al. (2) as a discrete, hypersensitive nodule within a palpable taut band of skeletal muscle that, when compressed, elicits a local twitch response—a brief, involuntary contraction of the affected muscle fibers—and a recognizable pattern of referred pain distant to the compression site. MTrPs are classified as active when they produce spontaneous pain at rest or during movement, and as latent when they are painful only on direct compression.

Active and latent MTrPs share the same fundamental neurophysiological substrate—motor endplate dysfunction, taut band, and local twitch response—and the same motor, sensory, and autonomic features; they differ primarily in (i) the presence of spontaneous pain, (ii) a lowered mechanical nociceptive threshold (MNT), and (iii) the local biochemical changes documented at active sites (3, 6, 7). Despite this shared substrate, the clinical consequences differ: active MTrPs directly contribute to pain and gait dysfunction, while latent MTrPs alter muscle activation patterns and predispose affected muscles to fatigue and injury even in the absence of spontaneous pain (3, 6).

MTrPs are associated with a characteristic cluster of findings on examination: a palpable taut band, local tenderness, a local twitch response to snapping palpation, referred pain in a characteristic distribution, and associated autonomic phenomena (piloerection, local sweating) without a fixed topography, and reduction in range of motion of the affected muscle and its associated joints (2, 3). In horses, the local twitch response—a visible fasciculation of a small muscle region under the skin on palpation—is an identifying feature that distinguishes MTrPs from diffuse muscle soreness or myopathies (8). However, eliciting and reliably interpreting the local twitch response (and palpatory MTrP diagnosis in general) is a highly specialised clinical skill: it requires snapping palpation or dry-needle insertion of the taut band, and the cited human studies document substantial inter-examiner variability when standardised training is absent (9).

### 2.2 The energy crisis model of trigger point formation

The most widely accepted mechanistic model of MTrP formation is the integrated hypothesis, first proposed by Simons and subsequently extended by multiple authors (2, 10–14). The model proposes that MTrPs originate from dysfunctional motor endplates at which excessive acetylcholine release produces persistent sarcomere contracture—localized contraction that does not depend on action potentials and therefore cannot be resolved by normal neural inhibition (11, 12). This sustained sarcomere shortening increases local metabolic demand while simultaneously compressing local microvasculature, reducing oxygen delivery and producing local ischemia. The resulting energy crisis leads to accumulation of nociceptive sensitizing substances including substance P, bradykinin, serotonin, norepinephrine, tumor necrosis factor-alpha (TNF-α), and prostaglandins within the trigger point microenvironment (15, 16). Gerwin’s most recent unified theory (14) further proposes a failure of pre- and post-synaptic feedback control mechanisms at the dysfunctional endplate as the underlying driver, providing a mechanistic update to the original integrated hypothesis.

Shah and colleagues (15, 16) used *in vivo* microdialysis at human MTrPs to directly measure the biochemical milieu and demonstrated significantly elevated concentrations of protons (pH 4.7–5.0), bradykinin, substance P, calcitonin gene-related peptide (CGRP), and TNF-α at active MTrP sites compared with latent MTrPs or normal muscle. This acidic, nociceptive biochemical environment activates and sensitises local group III (A-delta) and group IV (C-fiber) muscle afferents, producing spontaneous pain and hyperalgesia. Critically, the same sensitized afferents also respond to innocuous mechanical stimuli such as muscle stretch or contraction, meaning that normal locomotor loading of a muscle containing active MTrPs generates nociceptive input that, in the horse, may manifest as reluctance to engage, shortened stride, stiffness, or, in severe cases, overt lameness. The translational relevance of these biomarker findings to equine medicine is considerable: substance P and CGRP, the principal neuropeptides driving peripheral sensitization at the MTrP, are measurable in equine serum and synovial fluid.

Their elevation has been documented in human subjects with chronic musculoskeletal pain (15, 16); equine-specific microdialysis studies at MTrP sites have not yet been performed. The conservation of nociceptive neurochemistry across mammalian species makes it likely that a comparable pro-nociceptive milieu characterizes equine MTrPs—an assumption supported by the electrophysiological findings of Macgregor and von Schweinitz (5).

### 2.3 Peripheral and central sensitization

The sensitized nociceptors at active MTrPs undergo further pathological changes through peripheral sensitization: the activation threshold of group III/IV afferents is progressively reduced, such that stimuli that would normally be sub-threshold come to elicit pain responses (3, 10). In horses, pressure algometry studies—measuring mechanical nociceptive thresholds (MNTs) of epaxial muscles using calibrated algometry—have documented significantly reduced MNTs in horses with clinical back pain compared with healthy controls (17). Reduced MNTs indicate peripheral sensitization of muscular nociceptors consistent with active MTrP biology (18).

With chronicity, peripheral sensitization progresses to central sensitization: spinal dorsal horn neurons become hyperexcitable, their receptive fields expand, and the threshold for activation decreases (19). Central sensitization produces allodynia and secondary hyperalgesia remote from the primary MTrP site (10, 20). In the horse, central sensitization from chronically active MTrPs may explain the clinical observation of diffuse, poorly localized pain responses during lameness examination that do not correspond to single anatomical structures and that do not resolve with targeted perineural blocks—a presentation commonly attributed to ‘poor performance’ or ‘behavioral issues’ rather than recognized as a pain syndrome (21, 22).

### 2.4 Active and latent trigger points: the clinical activity spectrum

The classification of MTrPs into active and latent states represents a clinically important continuum of trigger point activity (23); sites without identifiable taut bands or local twitch responses are designated as *absent* rather than “inactive.” Ge and Arendt-Nielsen (23) define latent MTrPs as foci of hyperirritability within a taut band that produce pain and autonomic responses only on direct mechanical stimulation, not spontaneously, but that nonetheless generate measurable motor dysfunctions independent of pain. Electromyographic studies in human subjects confirm that latent MTrPs significantly alter the temporal activation patterns of synergist and antagonist muscles, increasing co-contraction and reducing movement efficiency even in the complete absence of reported pain (6, 23). In sport horses, this distinction has direct clinical relevance: a horse may harbor numerous latent MTrPs that do not produce overt lameness yet may systematically degrade movement quality, reduce collection capacity, and predispose affected muscles to transition to the active state under increased training load, fatigue, or thermal stress.

Because absolute differentiation between active and latent MTrPs cannot rely on patient-reported pain in non-verbal species, the classification in horses is operational and is made on the basis of converging objective indicators: (i) the presence of spontaneous pain-related behavior at rest or during work—assessed using validated equine pain assessment tools such as the Ridden Horse Pain Ethogram (24, 59), the Horse Grimace Scale (60), and the Equine Pain Face (61)—versus pain provoked only on direct mechanical stimulation; (ii) significantly lower mechanical nociceptive thresholds at active compared with latent MTrP sites on pressure algometry (15, 17, 23); (iii) the differential pattern of spontaneous electrical activity at active versus latent sites on needle electromyography, which represents the only directly equine-validated electrophysiological differentiator (5, 12); and (iv) the intensity of the local twitch response and defensive (“jump sign”) reaction on snapping palpation (3). The authors acknowledge that this classification remains operational rather than absolute and that some overlap between activity states is unavoidable in non-verbal species; this limitation should be taken into account when interpreting clinical findings.

**Figure 1:**
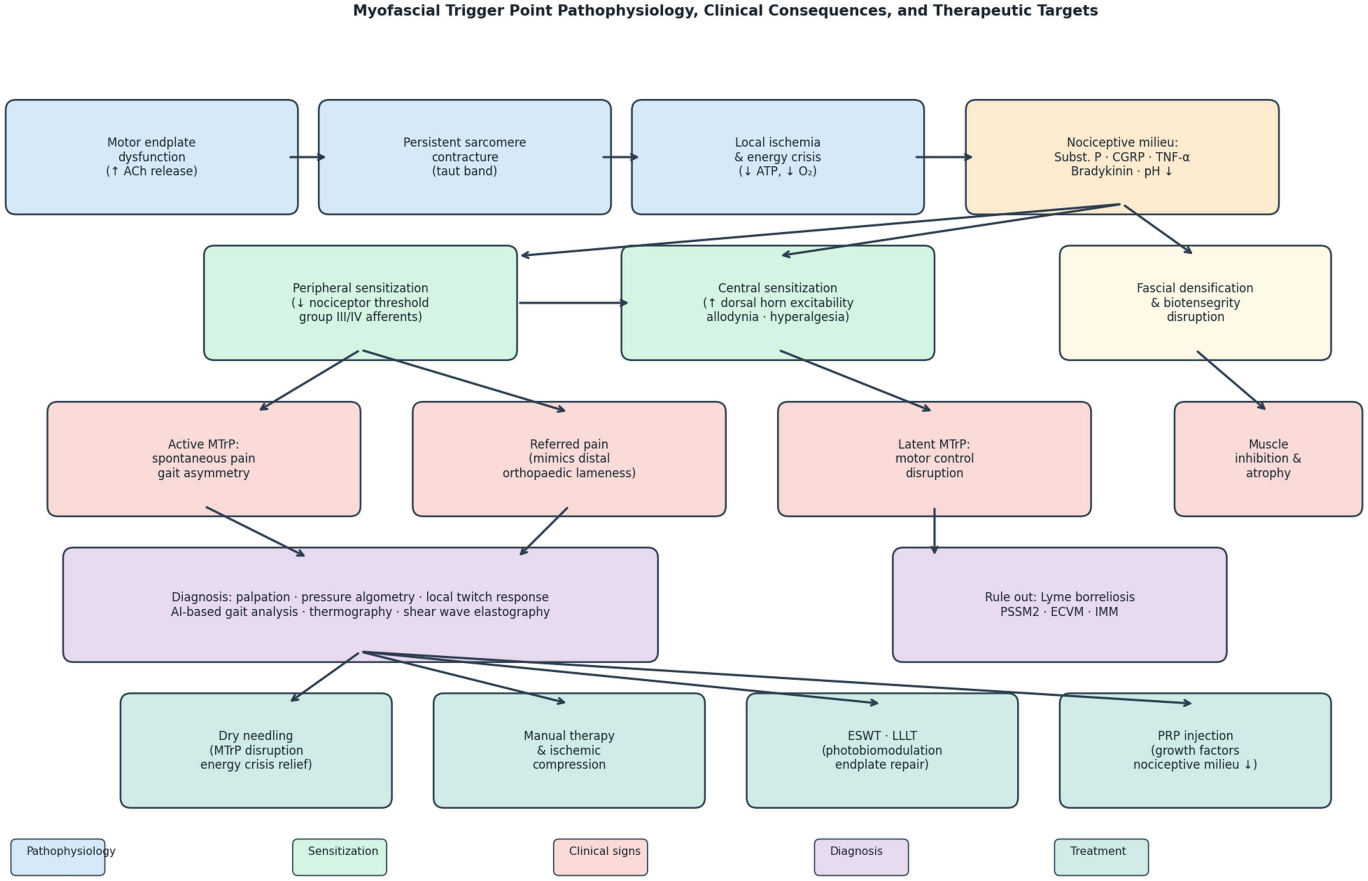
Schematic overview of myofascial trigger point (MTrP) pathophysiology, clinical consequences, and therapeutic targets. Motor endplate dysfunction initiates a self-perpetuating energy crisis that releases nociceptive mediators (substance P, CGRP, TNF-α, bradykinin) leading to peripheral and central sensitization. Clinical manifestations include active MTrPs with spontaneous pain, referred pain mimicking orthopaedic lameness, latent MTrPs with covert motor control disruption, and fascial biotensegrity disturbance. Diagnostic and therapeutic intervention points are shown. ACh, acetylcholine; CGRP, calcitonin gene-related peptide; TNF-α, tumor necrosis factor-alpha; ESWT, extracorporeal shock wave therapy; LLLT, low-level laser therapy; PRP, platelet-rich plasma; PSSM2, polysaccharide storage myopathy type 2; ECVM, equine complex vertebral malformation; IMM, immune-mediated myositis.

After resolution of an active MTrP—through treatment, rest, or spontaneous resolution—the predisposing factors that originally generated the lesion frequently persist. These residual factors fall into three categories: (1) *structural changes at the motor endplate*, in particular increased miniature endplate potential frequency, persistent low-grade acetylcholine leakage, and local sarcomere shortening, all of which lower the threshold for renewed sustained contracture under nociceptive or mechanical drive (12, 25); (2) *fascial tissue remodelling*, including densification of the loose connective tissue surrounding the affected muscle fibers, increased collagen cross-linking, and reduced inter-layer gliding, which alter local force transmission and predispose to recurrent overload (26, 27); and (3) *biomechanical drivers* such as repetitive sport-specific overload, ill-fitting saddles, poor hoof balance, and underlying orthopaedic asymmetries, which continue to apply the same mechanical stress that originally provoked the lesion (28–30). Within the binary active/latent framework, these residual factors translate into an elevated recurrence risk: under conditions of mechanical overload, systemic inflammation, or nociceptive sensitization from adjacent active MTrPs or joint pathology, latent MTrPs may transition back into the active state (2, 25). Shah et al. (25) specifically demonstrated that the transformation between latent and active states is driven by the same biochemical cascade (acidification, accumulation of substance P, CGRP, bradykinin, and TNF-α), differing only in the magnitude and persistence of nociceptive mediator accumulation. The clinical implication for equine practice is that MTrP treatment alone is rarely sufficient: it must be combined with systematic management of the predisposing mechanical, nutritional, training, and tack-related factors—a rehabilitation strategy currently absent from most equine sports medicine protocols (Figure 1).

### 2.5 Differential diagnoses in equine myofascial lameness: Lyme borreliosis and primary myopathies

Myofascial pain syndrome (MPS), as defined in the literature, is characterised by the presence of discrete MTrPs; presentations without identifiable taut bands or local twitch responses do not fulfill the diagnostic criteria for MPS and are better classified as differential diagnoses in the equine lameness workup. The most clinically important differential is *Borrelia burgdorferi* infection (Lyme disease), which represents one of the most clinically significant infectious causes of diffuse muscle pain in horses in endemic regions. Divers et al. (31) documented that the most common musculoskeletal manifestations of confirmed Borrelia infection include generalised muscle stiffness, diffuse myalgia, multi-limb wandering lameness, and hyperesthesia to palpation—a pattern that does not conform to discrete MTrP topography [i.e., the spatial distribution of taut bands and twitch responses within an individual muscle body, as mapped anatomically by Simons et al. (2)] but reflects widespread nociceptor sensitization of muscular tissue by the spirochetal infection and associated immune-inflammatory response.

The diagnostic distinction between MPS-associated lameness (driven by discrete MTrPs) and Lyme-associated diffuse myalgia (driven by systemic spirochetal infection) is clinically critical, and the two conditions can be comorbid: chronic Lyme-associated nociceptor sensitization may favor secondary MTrP formation in overloaded muscle groups. Horses with Lyme-associated myalgia will not respond predictably to MTrP-specific interventions such as dry needling of individual taut bands, because the primary nociceptive driver is systemic infection rather than localised endplate dysfunction. In endemic regions, serological screening should therefore be incorporated into the diagnostic algorithm for any horse presenting with unexplained multi-limb stiffness, diffuse muscle hyperalgesia, or generalised performance decline before attributing the presentation to MPS. In practical terms this means that when myofascial palpation reveals diffuse hyperalgesia without discrete taut bands or twitch responses, particularly in horses living in or returning from endemic regions, a combined *Borrelia* serology panel (C6 ELISA together with a multiplex SNAP or Lyme Multiplex assay) should precede or accompany myofascial therapy. A positive serological result does not, on its own, prove clinical Lyme disease, but in this clinical context it shifts the working diagnosis from MPS to Lyme-associated myalgia (with or without secondary MTrPs) and redirects the treatment plan toward antibiotic therapy in addition to any indicated myofascial intervention.

A further clinically important differential within the spectrum of equine muscle pain disorders is primary muscle disease. In veterinary medicine, muscle pathology has historically been recognised only when accompanied by markedly elevated serum muscle enzymes (AST, CK) or diagnostic muscle biopsy findings—the far end of the clinical spectrum. Horses with polysaccharide storage myopathy type 2 (PSSM2) and myofibrillar myopathy (MIM) present with altered muscle fiber architecture and energy metabolism that predisposes to MTrP formation through the same energy crisis mechanism, but also produce diffuse myalgia, muscle stiffness, and exercise-induced dysfunction in the absence of discrete trigger point topography (32). Similarly, immune-mediated myositis (IMM) and vitamin E–selenium deficiency myopathy can produce generalised muscle hyperalgesia that mimics or co-exists with MTrP pathology. The clinical argument advanced in this review is that these primary myopathies represent one end of a continuum, while MPS represents the more prevalent and systematically underdiagnosed other end, and that the diagnostic algorithm must be capable of distinguishing between them.

A related area of clinical relevance is the documented spatial overlap between acupuncture point and MTrP locations. Studies in horses have demonstrated that reactive acupuncture point sensitivity—assessed by systematic scanning of known acupoint locations—correlates with the distribution of musculoskeletal lesions (33, 34); the cited references address musculoskeletal lesion localisation rather than myofascial pain *per se*, and we have therefore removed any direct equivalence between acupoint reactivity and myofascial pain. Alfaro (34) reported a significant correlation between acupuncture point reactivity and the anatomical location of confirmed lesions in a large equine cohort, and Schmid and Aebischer (33) proposed a sensitivity scanning protocol for clinical use; we cite their work as a clinical scanning approach rather than as a validated MTrP diagnostic standard. Importantly, MTrP diagnosis rests on palpatory criteria (taut band, local twitch response, pain on compression, behavioral indicators of referred pain). Pressure algometry provides an objective measure of mechanical pain threshold and can be used for treatment-response monitoring; acupuncture point scanning may assist spatial localisation; neither replaces palpatory diagnosis (9, 35).

## 3 Evidence for myofascial trigger points in horses

### 3.1 Electrophysiological confirmation

The most rigorous evidence for equine MTrPs comes from electrophysiological study. Macgregor and von Schweinitz (5) used needle electromyography (EMG) to record spontaneous electrical activity at clinically identified MTrP sites in the equine cleidobrachialis muscle. MTrP sites demonstrated characteristic spontaneous electrical activity—a combination of low-amplitude end-plate noise and high-amplitude spike discharges—identical to the spontaneous electrical activity documented at MTrPs in human and rabbit muscle (12). Control sites within the same muscle showed no spontaneous electrical activity. This study established that equine MTrPs are not merely clinical impressions but electrophysiologically defined entities with the same pathophysiological substrate as MTrPs documented in human and laboratory animal models (5, 12).

### 3.2 Prevalence in sport horses

A recent study comparing trigger point prevalence in dressage and show-jumping horses (28) found MTrPs in all examined animals, with prevalence exceeding 60% in the thoracolumbar musculature across both disciplines. Dressage horses showed higher MTrP prevalence in the cervical musculature, particularly the semispinalis capitis and brachiocephalicus, consistent with the greater demands placed on neck positioning and collection in that discipline. Show-jumping horses showed higher prevalence in the thoracolumbar and gluteal regions, consistent with repeated jumping effort demands on hindlimb propulsion and landing absorption. The cohort in this study was small (*n* = 14), so the prevalence figures should be regarded as indicative rather than population-representative pending larger-scale equine surveys.

A related study of MTrPs in equine pectoral muscles (29) identified a significant association between active MTrPs in the pectoral musculature and girth-aversion behavior—a clinically common and frequently misattributed presentation in sport horses. The resolution of girth-aversion behavior following MTrP treatment in a subset of horses confirmed a direct causal relationship between active MTrPs and observable pain-related behavior, rather than a secondary association.

### 3.3 MTrPs and epaxial muscle dysfunction

The spinalis, longissimus, and iliocostalis lumborum are among the most commonly affected epaxial muscles in horses with back pain (8, 17). Dyson et al. (24) documented a significant association between epaxial muscle hypertonicity or pain on palpation and aberrant tack-up and mounted behavior in a population of sport horses, including girthiness, resistance to mounting, tail-swishing, and reluctance to engage hindquarters. A proportion of these horses had no radiographic or ultrasonographic abnormalities of spinal structures; this is consistent with a possible myofascial contribution to discomfort in these cases, although the cited dataset does not allow myofascial pain to be inferred as the primary or sole source.

Haussler (30) demonstrated that horses with thoracolumbar pain assessed by pressure algometry showed significantly reduced range of motion in lateral bending and dorsoventral flexibility compared with pain-free controls. This restriction of motion is consistent with the known effect of active MTrPs on joint range of motion in human patients: MTrPs produce sustained muscle shortening that mechanically limits joint excursion, reduces proprioceptive feedback accuracy, and alters the reflex muscle activation patterns required for coordinated locomotion (2, 3).

## 4 Referred pain from myofascial trigger points: the diagnostic trap

### 4.1 Mechanism of referred pain

The most diagnostically consequential feature of active MTrPs is their capacity to produce referred pain: spontaneous or provoked pain perceived at a site distant from the MTrP itself, in a characteristic distribution derived empirically from clinical observation in human patients [notably the systematic mapping by Simons et al. (2)]; these patterns may partially overlap with, but do not strictly follow, myotomal, sclerotomal, or dermatomal nerve distributions (2, 3, 10). The mechanism involves central sensitization of spinal dorsal horn neurons: convergent inputs from MTrP-derived nociceptors and from normally sub-threshold afferents innervating remote structures activate wide-dynamic-range (WDR) neurons in the dorsal horn, producing the perception of pain at the remote site despite the absence of local nociceptive input there (10, 20).

In human patients, MTrP-referred pain from proximal muscles is well documented to mimic peripheral joint pathology. Gluteus minimus MTrPs reproduce sciatica and lateral knee pain; iliopsoas MTrPs mimic hip joint pain; infraspinatus MTrPs mimic shoulder joint pathology (2). The clinical implication is direct: pain perceived in a distal structure does not confirm that the pathology is located there.

### 4.2 Referred pain patterns in equine lameness

Applying the established referred pain patterns of human myofascial anatomy to the equine musculoskeletal system may yield clinically testable predictions. MTrPs in the gluteus medius—a large, frequently overloaded hindquarter muscle with high prevalence in sport horses (28)—may project referred pain caudodistally into the hindlimb, potentially producing apparent hindlimb lameness with stifle or hock involvement (36, 37); the equine references (36, 37) document distal hindlimb lameness associated with proximal hindquarter pathology rather than direct referred pain mapping, and we therefore qualify the projection as an extrapolation from anatomical homology with the human gluteus medius referral zone. MTrPs in the biceps femoris and semimembranosus may project distally and cranially, potentially mimicking gastrocnemius or tarsal pain. MTrPs in the longissimus lumborum at the thoracolumbar junction may contribute to girthiness, lateral bending resistance, and apparent cranial thoracic pain on mounting, consistent with the clinical observations reported by Dyson et al. (38), though direct referred pain mapping has not been validated in horses.

The mechanistic premise that gluteal MTrPs can produce apparent distal hindlimb signs is established primarily in the human myofascial pain literature, in which the resolution of distal referred pain (lateral thigh, calf, and knee region) following targeted treatment of gluteus medius and gluteus minimus MTrPs is well documented (2, 7). Direct equine mapping studies of distal sign resolution after gluteal MTrP treatment have not been published. Indirect equine support comes from observational reports of distal hindlimb sign improvement following treatment of the proximal hindquarter region (37) (although that report was confounded by concurrent hoof rebalancing) and from objective AI-based gait analysis demonstrating distal kinematic improvement after dry needling of proximal MTrPs (39); both should be regarded as hypothesis-generating rather than confirmatory in the equine context.

The clinical consequence is a diagnostic trap: when a horse presents with apparent hindlimb lameness—reduced hindlimb swing, shortened caudal phase, positive response to hock flexion test—the standard workup proceeds to intraarticular anesthesia of the distal tarsal joints. If this produces partial improvement (as it may, through systemic analgesic effects or placebo), the lameness is attributed to distal tarsal osteoarthritis and treated accordingly. The proximal MTrPs generating the referred hindlimb pain are not identified, not treated, and not resolved. The horse may show temporary improvement before returning to the same clinical presentation—a pattern in which the myofascial origin may remain unrecognized and untreated.

### 4.3 Why perineural blocks do not identify myofascial pain

Perineural anesthesia blocks afferent signals from the anatomical territory distal to the injection site (40). It may not fully interrupt nociceptive input from the muscle or myofascia proximal to the block. When referred pain originates from a proximal MTrP via central sensitization mechanisms, blocking distal afferents may not interrupt the primary nociceptive input; the dorsal horn neurons remain activated by the proximal MTrP afferents and continue to generate referred pain perception. A perineural block performed distal to the referred pain site will therefore not consistently eliminate pain of myofascial origin—and the absence of response to distal nerve blocks is frequently—and incorrectly—interpreted as indicating that the pain source is ‘not in that region’ rather than that it is proximal and myofascial (3, 8).

This limitation of the standard diagnostic algorithm has practical consequences for diagnostic accuracy. A horse with active gluteal MTrPs producing referred hindlimb pain will not show consistent improvement to low palmar nerve block, nor to tibial and fibular nerve block, nor necessarily to fetlock or coffin joint anesthesia—because none of these blocks interrupt the proximal myofascial nociceptive drive. The lameness may be classified as ‘high hind’ and proceeds to radiography of the stifle or hip, with imaging often revealing either normal anatomy or age-appropriate incidental findings that may be attributed diagnostic significance beyond their clinical relevance (41).

## 5 Biomechanical consequences of myofascial trigger points on equine gait

### 5.1 Muscle inhibition and altered activation patterns

Active MTrPs alter muscle function through two mechanisms beyond pain: direct mechanical shortening of the affected muscle fibers due to the sustained sarcomere contracture of the taut band, and reflex inhibition of the affected muscle mediated through the nociceptive input reaching the spinal cord (2, 3, 6). The combination of shortened resting length (2), reduced extensibility (3, 30), and inhibited voluntary contraction—documented electromyographically in human subjects (6)—produces a characteristic pattern: the affected muscle is simultaneously shortened (limiting joint range of motion in the direction of elongation), weakened in its contractile capacity, and hypertonic on palpation. In horses, this pattern can be assessed by systematic palpation of major locomotor muscles as part of the lameness examination, although reliable detection requires examiner training in standardised palpation technique (9).

Electromyographic studies in human subjects demonstrate that MTrPs in a given muscle alter the temporal activation pattern of synergists and antagonists through altered proprioceptive feedback and spinal reflex reorganization (3, 6). The practical implication is that MTrPs in one muscle do not merely affect that muscle in isolation: they reorganize the motor control strategy for the entire functional movement pattern in which that muscle participates (i.e., compensatory lameness). In horses, this could mean that a single active MTrP in the gluteus medius does not merely cause local pain—it may alter hindlimb protraction timing, stifle flexion amplitude, hindquarter engagement, and the reflex-mediated stabilization of the thoracolumbar junction, potentially producing a multisegmental gait disturbance that may not localize clearly to one anatomical structure on standard lameness examination; such compensatory multisegmental adaptation patterns are described in lame horses generally (42), although MTrP-specific kinematic data in horses remain to be established.

### 5.2 Kinematic and kinetic gait signatures of MTrP-associated lameness

Throughout this review the term *MTrP-associated lameness* is used as a descriptive construct for gait dysfunction in which discrete MTrPs are the dominant identifiable nociceptive driver. It is not yet an established diagnostic entity in the veterinary literature and is introduced here as a working clinical concept; further validation in controlled equine studies is required.

Horses with primary MTrP-associated lameness may present with a constellation of kinematic findings that differ qualitatively from those of joint- or tendon-origin lameness. The following biomechanical features, derived from clinical observation in horses and from documented effects of MTrPs on gait in human and animal subjects [e.g., altered hip and trunk kinematics in human patients with gluteal and lumbar MTrPs (18, 23), altered fore- and hindlimb loading in canine myofascial pain (4)], may characterize MTrP-associated gait disruption and may assist differential diagnosis:

First, the gait asymmetry in MTrP-associated lameness is typically warm-up-dependent: it is most pronounced after rest or in cold temperatures (when ischemia in the trigger point microenvironment is enhanced), and it decreases substantially after 10–20 min of active exercise as increased local blood flow partially relieves the energy crisis sustaining the MTrP (8). Joint-origin lameness, by contrast, may worsen with continued loading (43).

Second, MTrP-associated lameness may present with asymmetric muscular development—atrophy of inhibited muscles and compensatory hypertrophy of synergists that have assumed additional load—visible and palpable on topographic examination; however, such patterns may also be observed in other conditions, including primary myopathies, disuse, or systemic muscle disorders (30). This pattern of muscular asymmetry may be disproportionate to or inconsistent with the localization suggested by nerve block results, warranting consideration of myofascial involvement.

Third, the kinetic signature of myofascial hindlimb lameness may differ from joint-origin lameness in push-off mechanics. MTrP-induced inhibition of gluteal or hamstring musculature is hypothesised to reduce active propulsive push-off impulse—not through pain-related guarding of joint loading, but through reduced recruitment of the affected muscle during the late stance phase. Equine kinetic data supporting this specific MTrP-related mechanism are limited; Weishaupt et al. (44) documents the broader effect of head and neck position on vertical ground reaction forces in dressage horses, which is consistent with—but does not directly establish—an MTrP-specific reduction in propulsive impulse. The pattern is therefore best regarded as an empirical extrapolation from human biomechanics requiring prospective equine validation. The proposed signature would be a reduced vertical impulse and shortened caudal phase of the stride without the characteristic loading-phase asymmetry seen in distal orthopaedic lameness; objective kinetic analysis using force platforms or instrumented horseshoes may help to characterise these patterns, although similar gait signatures can be observed in distal orthopaedic lameness, and careful interpretation is required.

### 5.3 Myofascial pain and collection deficits in dressage horses

In dressage horses, MTrPs in the epaxial and hindquarter musculature may produce specific limitations on collection capacity that are biomechanically distinct from training limitations or articular restrictions. The hallmark of correct collection—increased hindlimb engagement, caudal shift of the center of mass, and maintenance of impulsion and rhythm—requires coordinated, uninhibited contraction of the gluteal muscles, hamstrings, and psoas group during the stance phase, combined with unrestricted lengthening of these muscles during the swing phase (45, 46).

Active MTrPs in any of these muscle groups may produce a dual disruption: reduced contractile capacity during the propulsive phase (decreased push-off and hindlimb engagement) and restricted lengthening during the swing phase (shortened caudal stride length and reduced hindlimb flexion). The clinical presentation may include a horse that appears unwilling or unable to engage its hindquarters, may show lateral tail deviation (from asymmetric lumbar MTrP tension), may demonstrate resistance to lateral bending in the direction of the affected side, and may show reduced suspension in collection exercises such as piaffe and canter pirouette—a potential biomechanical consequence of MTrP-induced reduction in push-off impulse.

### 5.4 Biotensegrity, fascial force transmission, and myofascial pain

The conventional myofascial pain model conceptualizes MTrPs as discrete, localized dysfunctions within individual muscles. A more comprehensive mechanobiological framework—biotensegrity—provides a structural explanation for the regional or systemic effects of single MTrPs that cannot be fully accounted for by neurological referred pain mechanisms alone (47). Biotensegrity, as formalized by Levin and subsequently applied to fascial biomechanics by Bordoni and Myers (47), models the musculoskeletal system as a continuous tensional network in which fascial membranes, ligaments, and intermuscular septa transmit tensional force globally across the entire body. Within this framework, fascia is not merely a passive connective tissue envelope but an active force-transmitting and mechanosensory network whose tensional state is modulated by the contractile activity of embedded myofibroblasts and regulated by mechanoreceptive fibroblasts capable of responding to mechanical deformation with altered collagen synthesis and matrix remodeling.

Fascial dysfunction develops through a progression of tissue changes that can be described as densification, adhesions, and fibrosis (26). Densification is characterized by viscoelastic change within the loose connective tissue without histological alterations and is typically associated with chronic, repetitive use injuries such as those common in competitive sport horses. Adhesions involve histological changes in both loose and dense connective tissues and are usually associated with trauma or early surgical healing phases. Fibrosis represents the end stage: histological changes within the dense connective tissue resulting from chronic tissue injury or inflammation. Each stage progressively impairs the force-transmitting and gliding properties of the fascial network, amplifying the mechanical consequences of MTrP-associated muscle tension and further impairing the global biotensegrity system. Early identification and treatment of MTrPs—before fascial densification progresses to adhesion or fibrosis—is therefore not only a pain management strategy but a tissue preservation strategy.

Within the biotensegrity model, an active MTrP—which produces a localized zone of sustained high tension in the taut band and surrounding fascial sheaths—alters the local, regional, and global tensional distribution of the fascial network (47). Because equine thoracolumbar and gluteal fasciae are anatomically continuous structures, a single active MTrP in the gluteus medius may increase fascial tension in the thoracolumbar fascia, the sacrotuberous ligament complex, and the contralateral hindlimb fascial network simultaneously (48)—a mechanistic inference requiring prospective biomechanical validation—producing loading asymmetries across multiple regions without the anatomical specificity implied by single-muscle models. This global tensional redistribution accounts for the clinical observation that horses with primary MTrP-associated lameness frequently present with widespread palpation tenderness, diffuse topographic asymmetry, and multi-regional movement restriction—a presentation more consistent with fascial network disruption than with isolated muscle pathology. The clinical implication is that comprehensive myofascial assessment in horses must evaluate the entire fascial network—cervical, thoracolumbar, pelvic, and appendicular—rather than individual muscles in isolation (26), and that treatment of a single dominant MTrP may rapidly relieve tension in remote fascial regions, explaining the occasional observation of rapid and disproportionately extensive gait improvement following treatment of a single trigger point. Clinical integration of fascial assessment protocols into equine rehabilitation therefore requires systematic evaluation of the entire myofascial system, and manual or instrument-assisted techniques targeting fascial layers have demonstrated measurable improvements in tissue mobility and performance parameters (27).

## 6 Clinical, diagnostic, and therapeutic implications

### 6.1 Integrating myofascial examination into the lameness workup

A systematic myofascial examination requires no specialist equipment and adds approximately 10–15 min to a standard lameness assessment; this estimate is based on bilateral palpation of approximately 12–15 standardised muscle groups (cervical, pectoral, epaxial, gluteal, hamstring, and proximal forelimb) at roughly 30–60 s per group. The examination assesses, in sequence: (1) topographic inspection for muscular asymmetry and atrophy; (2) palpation of major locomotor muscles for taut bands and local twitch responses—the primary diagnostic step for MTrP identification; (3) range of motion testing of cervical, thoracolumbar, and appendicular joints for MTrP-related restriction; and (4) where available, algometric assessment of mechanical nociceptive thresholds at standardised sites. Pressure algometry is included as an objective, quantitative measure of pain threshold and treatment response rather than as a primary diagnostic criterion for MTrP identification; the diagnostic decision remains palpatory (8, 17, 30, 35).

The most diagnostically productive muscles to examine in horses with suspected MTrP-associated lameness are, in order of clinical prevalence: gluteus medius, longissimus lumborum (L1–L5 region), hamstring musculature (biceps femoris, semimembranosus, semitendinosus), brachiocephalicus, infraspinatus, triceps brachii, and the pectoral girdle (24, 28, 29). The identification of active MTrPs in these muscles is supported by the local twitch response and by behavioral evidence of provoked referred pain. Because horses are non-verbal, referred pain cannot be confirmed verbally and is necessarily inferred from indirect clinical surrogates: (i) the spatial distribution of behavioral pain responses (ear pinning, guarding, head turning, tail-swishing) elicited by systematic compression of the suspected MTrP, mapped against the expected referred zone; (ii) resolution of distal clinical signs following proximal MTrP treatment; and (iii) anatomical homology with documented human referred pain patterns. Referred pain identification in horses should therefore be regarded as an indirect clinical inference requiring prospective validation, rather than as a confirmed clinical sign. The myofascial examination should be considered alongside the standard nerve block protocol, rather than reserved for cases in which perineural anesthesia fails.

### 6.2 Treatment response as diagnostic confirmation

A distinctive and clinically useful feature of MTrP-associated lameness is the speed of response to targeted MTrP treatment. Dry needling of active MTrPs in horses produces measurable reductions in local tenderness and improvements in gait symmetry within 24–48 h in responsive cases (39, 49). This rapid response is mechanistically explained by the disruption of the sustained sarcomere contracture by needle insertion, the release of the energy crisis sustaining the trigger point, and the washout of the accumulated nociceptive biochemicals from the trigger point microenvironment through restored local perfusion (15, 16).

The practical diagnostic implication is that a trial of MTrP treatment—typically combining dry needling, manual pressure release, and/or percutaneous electrical needle stimulation (PENS) of identified active MTrPs—constitutes a legitimate and informative diagnostic approach in horses presenting with lameness of unclear origin or lameness that has not resolved consistently with conventional orthopaedic treatment. Rapid and sustained gait improvement following such treatment is supportive evidence of myofascial origin. The 3–5 session response window suggested in the equine clinical literature (8) reflects expert clinical consensus rather than evidence from controlled trials; it is provided as a working clinical guideline for when to broaden the orthopaedic workup if there is no response, and prospective controlled equine studies are required to validate both the optimal treatment combination and the response threshold.

### 6.3 Treatment of equine myofascial pain syndrome

Treatment of MPS in horses is multimodal and combines four therapeutic axes: (i) deactivation of the active MTrP, (ii) restoration of fascial mobility and muscle length, (iii) modification of the predisposing biomechanical and management factors, and (iv) supportive therapy of the local microenvironment. The principal modalities currently in clinical use are summarised below; with the exception of dry needling, the equine evidence base is largely extrapolated from human and small-animal data and is acknowledged as such throughout the review.

*Dry needling and PENS*. Dry needling—monofilament needle insertion directly into the taut band—is the most directly evidence-supported MTrP intervention in horses. Calatayud-Bonilla et al. (49) reported a prospective algometric controlled study showing increased mechanical nociceptive thresholds and reduced clinical pain scores after dry needling in horses with palpation-confirmed MTrPs, and Resano-Zuazu et al. (39) documented objective AI-based gait improvement after dry needling of proximal MTrPs. Treatment is typically delivered in 3–5 sessions at 5–14-day intervals, with one to four needles per MTrP; PENS adds low-frequency electrical stimulation (2–10 Hz) through the inserted needles and is used when sustained motor unit reset and analgesia are clinically required.

*Manual therapy: ischaemic compression and trigger point release.* Sustained digital compression of the MTrP for 30–90 s (“ischaemic compression”) and graded soft-tissue release techniques mechanically reduce taut band tension and facilitate post-treatment perfusion. In horses these techniques are usually combined with dry needling rather than used in isolation, and their efficacy in reducing palpation pain and increasing range of motion is supported by clinical case series and by the broader equine manual-therapy literature (27, 30).

*Stretching, controlled exercise, and rehabilitative loading.* Active and passive stretching of the affected muscle group, combined with progressive controlled exercise (long-and-low groundwork, in-hand mobilisation, hill work, cavaletti) restores muscle length and re-trains coordinated recruitment. The objective is to convert the mechanical relief produced by MTrP deactivation into a sustained change in motor pattern, thereby reducing recurrence risk (27, 30).

*Adjunctive physical modalities: ESWT, LLLT, thermotherapy, ultrasound.* Extracorporeal shock wave therapy (ESWT) delivered to MTrP sites produces defocused mechanical cavitation effects that disrupt the dysfunctional endplate region, reduce taut band stiffness, and improve local microcirculation; its non-invasive nature makes it particularly attractive in the equine setting (50). Low-level laser therapy (LLLT) at MTrP sites has shown efficacy in human randomised trials via mitochondrial photobiomodulation that restores ATP production in energy-depleted sarcomeres and reduces TNF-α and interleukin-1β (51). Local heat application, therapeutic ultrasound, and instrument-assisted soft-tissue mobilisation are commonly combined with the modalities above and serve to improve fascial gliding and accelerate post-treatment recovery (27).

*Regenerative options: PRP.* Platelet-rich plasma (PRP) injected into active MTrPs has been shown in randomised controlled trials in human patients to produce sustained reductions in pain and MTrP sensitivity superior to dry needling or corticosteroid injection (52, 53). Proposed mechanisms include growth factor-mediated motor endplate repair, local anti-inflammatory effects via transforming growth factor-*β*, and modulation of the nociceptive biochemical milieu. Targeted application of PRP to identified equine MTrPs represents a logical translational extension and should be evaluated in prospective equine trials.

*Pharmacological adjuncts.* Systemic NSAIDs may be used short-term for pain control during treatment escalation but do not deactivate the MTrP itself; muscle relaxants (e.g., methocarbamol) and anxiolytic/myorelaxant management are sometimes used in horses with severe paraspinal hypertonicity. Antibiotic therapy is reserved for confirmed Lyme-associated myalgia (see Section 2.5). Pharmacological agents are considered supportive rather than primary, and should not replace mechanical deactivation of the MTrP.

*Management and predisposing-factor correction.* Sustained therapeutic success requires correction of the predisposing factors identified in Section 2.4: saddle-fit assessment, dental and bridle-fit checks, hoof balance, training-load adjustment, and screening for primary myopathies (PSSM2, MIM, IMM) and ECVM. Without this corrective layer, treated MTrPs are at high risk of reactivation under continued mechanical drive.

Together, these modalities form the therapeutic backbone of equine MPS management. The evidence supporting each individual modality is uneven, and prospective, multicentre, controlled equine trials are required to define optimal modality combinations, treatment intervals, and responder profiles.

### 6.4 MTrPs as primary vs. secondary pathology: a diagnostic distinction

It is important to acknowledge that MTrPs frequently develop secondary to primary orthopaedic pathology: a horse with distal tarsal osteoarthritis may develop compensatory overloading of the ipsilateral gluteal and lumbar muscles, which can lead to secondary MTrP formation; this compensatory pattern is supported by equine kinematic and kinetic studies of compensatory loading strategies in lame horses (42) and by clinical pain biomechanics literature in other species. In this scenario, treating the MTrPs alone will not eliminate the primary joint pain, and the clinical response to MTrP treatment will be partial and temporary. The distinction between primary and secondary myofascial lameness is therefore a diagnostic priority that requires both myofascial examination and comprehensive orthopaedic assessment (3, 8).

We acknowledge that the resolution of clinical signs is methodologically distinct from a formal diagnostic criterion: an international Delphi consensus has defined diagnostic criteria for MTrPs based on examination findings (taut band, local twitch response, pain on compression, and recognition or reproduction of the patient’s symptoms) rather than on treatment response (35). We therefore propose that, in the equine setting, the complete and sustained resolution of presenting gait asymmetry following targeted MTrP treatment—without concurrent orthopaedic intervention and without evidence of structural pathology on imaging—be regarded as *supportive clinical evidence* of primary MTrP-associated lameness rather than as a stand-alone diagnostic criterion. This formulation is stringent and correctly so: it prevents over-attribution of lameness to myofascial causes when structural pathology is present. However, its stringency should not discourage the systematic investigation of myofascial pain as a primary diagnosis, particularly in cases where standard orthopaedic investigation has been unrewarding or where the clinical presentation does not fit a clean articular or tendinous pattern.

## 7 Discussion

The evidence reviewed here supports a fundamental reappraisal of the role of myofascial trigger points in equine lameness. MTrPs appear to be common in sport horses, although the available equine prevalence data are derived from small cohorts [e.g., 14 horses in Portier et al. (28)] and should be regarded as indicative pending larger surveys. They produce electrophysiologically confirmed nociceptive activity, may generate referred pain patterns that mimic distal orthopaedic pathology, can cause measurable biomechanical gait disruption through muscle inhibition and altered motor control, and respond rapidly and specifically to targeted treatment. The systematic exclusion of MTrPs from the equine lameness diagnostic algorithm is not justified by the available evidence and may result in diagnostic inaccuracy, inappropriate treatment, and preventable welfare compromise.

The primary obstacle to the integration of myofascial examination into equine practice is not evidence but convention. The orthopaedic model of lameness, with its technically sophisticated imaging modalities and financially productive injection protocols, dominates equine sports medicine in a way that leaves little diagnostic space for a clinical examination technique that requires trained hands, anatomical knowledge, and time rather than equipment. Overcoming this institutional inertia requires both the accumulation of additional equine-specific evidence—particularly controlled studies demonstrating gait improvement following MTrP treatment—and active education of equine clinicians in systematic myofascial palpation.

A further consideration is the interaction between myofascial pain and other conditions common in the authors’ clinical experience. Horses with PSSM2 (polysaccharide storage myopathy type 2) and MIM (myofibrillar myopathy) present with muscle pathology that predisposes to MTrP formation through the altered energy metabolism and structural integrity of affected fibers (32). Horses with congenital cervical vertebral anomalies (e.g., equine complex vertebral malformation, ECVM) develop secondary cervical and forelimb myofascial pain from aberrant joint mechanics and compensatory muscle loading (54). Horses with trigeminal-mediated headshaking show epaxial and cervical MTrPs in a distribution consistent with reflex muscle guarding from chronic trigeminal pain (55). In each of these conditions, the myofascial component contributes independently to the clinical presentation and may respond to targeted treatment, improving overall outcomes when included in the management protocol.

The welfare implications of systematic underdiagnosis of myofascial pain are considerable. A horse with chronically active MTrPs that produces apparent distal lameness may undergo repeated nerve block and injection protocols targeting structures that are not the primary pain source. Each unsuccessful treatment cycle delays effective care, accumulates iatrogenic tissue damage from repeated needle placement, and imposes ongoing pain on the animal. The inclusion of myofascial assessment as a routine component of lameness workup is therefore not merely a therapeutic adjunct but an ethical imperative.

## 8 Future perspectives

The field of equine myofascial pain research is nascent relative to its human medicine counterpart; however, recent advances across multiple domains suggest a rapidly evolving research landscape and several areas represent high-priority targets for future investigation (56). First, the development of objective, quantifiable biomarkers for MTrP activity in horses is essential for establishing the diagnostic validity required for broader clinical adoption. Pressure algometry, which already provides reproducible mechanical nociceptive threshold data (17), requires standardization of technique, site selection, and reference ranges across breeds and disciplines. In parallel, imaging-based biomarkers are gaining increasing attention. Shear wave elastography—a non-invasive ultrasound technique capable of quantifying muscle and fascial stiffness—has shown promise for MTrP identification in human medicine and warrants evaluation in horses. Recent studies have demonstrated high intra-examiner reliability of shear wave elastography for assessing muscle stiffness, although inter-examiner variability remains a limitation, highlighting the need for standardized protocols and operator training (57). Thermographic assessment of skin surface temperature overlying active MTrPs may provide an accessible clinical screening tool (27, 58), although its specificity and sensitivity remain to be established. Together, these approaches suggest a future diagnostic framework integrating clinical palpation with semi-quantitative and imaging-based biomarkers (56).

Second, artificial intelligence–based gait analysis represents a transformative technology for objectively quantifying the kinematic response to MTrP treatment. More broadly, advances in objective movement analysis are reshaping the evaluation of treatment response. Resano-Zuazu et al. (39) demonstrated that AI-based systems can detect subtle gait changes following dry needling in horses, providing objective evidence of treatment efficacy that manual assessment cannot reliably capture. Integration of such systems into prospective controlled trials of MTrP treatment would substantially strengthen the evidence base for myofascial lameness as a primary diagnosis, and may facilitate differentiation between myofascial and concurrent orthopaedic contributions to gait asymmetry.

Third, prospective evaluation of the therapeutic modalities summarised in Section 6.3—including PRP, ESWT, and LLLT applied directly to identified equine MTrP sites—is required to define optimal treatment parameters, identify responder profiles, and evaluate combined or multimodal treatment strategies in horses (50–53, 56).

Fourth, standardized training programmes in systematic equine myofascial palpation examination, analogous to the training standards that exist for other specialised examination techniques in equine practice, are necessary to allow the broad clinical implementation that the evidence base increasingly supports. The development of schematic models linking MTrP pathophysiology, biomechanical dysfunction, and therapeutic intervention may facilitate both research design and clinical interpretation (56).

Taken together, these findings suggest that emerging therapeutic strategies for MTrPs may be best understood not as isolated interventions but as components of a multimodal approach targeting different levels of MTrP pathophysiology. Mechanical disruption of the dysfunctional endplate region (i.e., dry needling, ESWT), modulation of the biochemical and inflammatory microenvironment (i.e., PRP), and restoration of cellular energy metabolism (i.e., LLLT) may act synergistically to reverse the energy crisis and sensitization processes underlying MTrP activity.

Finally, prospective, multicentre, controlled trials comparing myofascial diagnosis and treatment protocols with standard orthopaedic workup in horses with undefined lameness represent the highest priority for advancing the field, particularly when incorporating objective outcome measures such as biomechanical, imaging, and clinical parameters.

## 9 Conclusion

Myofascial trigger points are a clinically relevant and systematically underdiagnosed potential cause of equine lameness. The principal conclusions of this review are:

(1) MTrPs in horses are electrophysiologically confirmed entities with the same spontaneous electrical activity, biochemical microenvironment, and clinical characteristics as MTrPs documented in human and animal models.
(2) Active MTrPs may produce referred pain through central sensitization mechanisms, projecting apparent pain to sites distant from the trigger point. By analogy with the well-documented human literature, such referred pain may mimic distal orthopaedic lameness in horses and may not be consistently eliminated by perineural blocks distal to the referred site; direct experimental confirmation of MTrP-related referred pain mapping in horses is, however, not yet available, and the equine evidence remains indirect.
(3) In horses, MTrPs may cause gait asymmetry through muscle inhibition, altered motor control, and restricted range of motion. Proposed kinematic and kinetic signatures of equine MTrP-associated lameness—including warm-up-dependent improvement, asymmetric muscular development, and reduced push-off impulse without loading-phase asymmetry—are derived primarily from clinical observation in horses and from human comparative data; controlled equine studies are required to confirm these patterns and their differential value relative to joint- or tendon-origin lameness.
(4) The current standard equine lameness diagnostic algorithm does not include myofascial examination and cannot identify MTrPs as a pain source. This constitutes a systematic diagnostic gap with measurable welfare consequences.
(5) Systematic myofascial palpation examination should be integrated into routine equine lameness assessment as a first-tier diagnostic step. Rapid and sustained resolution of gait asymmetry following MTrP treatment, while not in itself a diagnostic criterion, should be regarded as supportive clinical evidence of primary myofascial origin.
(6) Treatment of equine MPS is multimodal and combines deactivation of the active MTrP (dry needling, PENS), restoration of fascial mobility and muscle length (manual therapy, stretching, controlled exercise), adjunctive physical modalities (ESWT, LLLT, thermotherapy), regenerative options (PRP), pharmacological adjuncts where indicated, and correction of the predisposing biomechanical and management factors. Prospective controlled equine trials are required to define optimal protocols.

These conclusions support a paradigm shift in equine lameness diagnosis from a purely orthopaedic model to one that integrates myofascial examination as a standard component of clinical assessment.
